# Comparison of qSOFA and SIRS for predicting adverse outcomes of patients with suspicion of sepsis outside the intensive care unit

**DOI:** 10.1186/s13054-017-1658-5

**Published:** 2017-03-26

**Authors:** Eli J. Finkelsztein, Daniel S. Jones, Kevin C. Ma, Maria A. Pabón, Tatiana Delgado, Kiichi Nakahira, John E. Arbo, David A. Berlin, Edward J. Schenck, Augustine M. K. Choi, Ilias I. Siempos

**Affiliations:** 1 Department of Medicine, Division of Pulmonary and Critical Care Medicine, New York-Presbyterian Hospital-Weill Cornell Medical Center, Weill Cornell Medicine, New York, NY USA; 2 Department of Medicine, Division of Emergency Medicine and Pulmonary Critical Care Medicine, New York-Presbyterian Hospital-Weill Cornell Medical Center, Weill Cornell Medicine, New York, NY USA; 3 First Department of Critical Care Medicine and Pulmonary Services, Evangelismos Hospital, University of Athens Medical School, Athens, Greece; 4 New York-Presbyterian Hospital-Weill Cornell Medical Center, Weill Cornell Medicine, 1300 York Avenue, New York, NY 10065 USA

**Keywords:** Infection, Critical care, Organ failure, Severe sepsis, Mortality

## Abstract

**Background:**

The Third International Consensus Definitions for Sepsis and Septic Shock (Sepsis-3) Task Force recently introduced a new clinical score termed quick Sequential (Sepsis-related) Organ Failure Assessment (qSOFA) for identification of patients at risk of sepsis outside the intensive care unit (ICU). We attempted to compare the discriminatory capacity of the qSOFA versus the Systemic Inflammatory Response Syndrome (SIRS) score for predicting mortality, ICU-free days, and organ dysfunction-free days in patients with suspicion of infection outside the ICU.

**Methods:**

The Weill Cornell Medicine Registry and Biobank of Critically Ill Patients is an ongoing cohort of critically ill patients, for whom biological samples and clinical information (including vital signs before and during ICU hospitalization) are prospectively collected. Using such information, qSOFA and SIRS scores outside the ICU (specifically, within 8 hours before ICU admission) were calculated. This study population was therefore comprised of patients in the emergency department or the hospital wards who had suspected infection, were subsequently admitted to the medical ICU and were included in the Registry and Biobank.

**Results:**

One hundred fifty-two patients (67% from the emergency department) were included in this study. Sixty-seven percent had positive cultures and 19% died in the hospital. Discrimination of in-hospital mortality using qSOFA [area under the receiver operating characteristic curve (AUC), 0.74; 95% confidence intervals (CI), 0.66–0.81] was significantly greater compared with SIRS criteria (AUC, 0.59; 95% CI, 0.51–0.67; *p* = 0.03). The qSOFA performed better than SIRS regarding discrimination for ICU-free days (*p* = 0.04), but not for ventilator-free days (*p* = 0.19), any organ dysfunction-free days (*p* = 0.13), or renal dysfunction-free days (*p* = 0.17).

**Conclusions:**

In patients with suspected infection who eventually required admission to the ICU, qSOFA calculated before their ICU admission had greater accuracy than SIRS for predicting mortality and ICU-free days. However, it may be less clear whether qSOFA is also better than SIRS criteria for predicting ventilator free-days and organ dysfunction-free days. These findings may help clinicians gain further insight into the usefulness of qSOFA.

**Electronic supplementary material:**

The online version of this article (doi:10.1186/s13054-017-1658-5) contains supplementary material, which is available to authorized users.

## Background

More than two decades ago, sepsis was defined as the combination of infection and Systemic Inflammatory Response Syndrome (SIRS) [1]. However, subsequent research revealed that sepsis is not an exclusively pro-inflammatory condition; rather, it may involve early anti-inflammatory responses [2]. Moreover, SIRS criteria were found to be too sensitive and insufficiently specific to identify infected patients at risk for a complicated course [3, 4]. In the light of such developments, the Third International Consensus Definitions for Sepsis and Septic Shock (Sepsis-3) Task Force recently redefined sepsis [5]. Sepsis is accordingly viewed as a “life-threatening organ dysfunction caused by a dysregulated host response to infection” [5]. Organ dysfunction was characterized by the acute increase of at least two points in the Sequential (Sepsis-related) Organ Failure Assessment (SOFA) score [5]. Given that SOFA requires laboratory testing and is rarely performed outside the intensive care unit (ICU), for patients in a non-ICU setting, the Sepsis-3 Task Force introduced a simpler algorithm, named quick SOFA (qSOFA) [5].

The qSOFA has merits according to its proponents. It is simple (consisting of three clinical elements, namely hypotension, tachypnea and altered consciousness), it can be easily and repeatedly assessed, it was generated through a data-driven approach, and in a large retrospective study it was more accurate than SIRS score for predicting death and ICU transfer of patients with suspected sepsis outside the ICU [6–8]. However, thoughtful criticisms have also been articulated. It has been stressed that the increased specificity of qSOFA over SIRS score for predicting poor prognosis may come at the expense of lower sensitivity, which may lead to delays in initiation of treatment [9]. Others pointed that it was not endorsed by key scientific societies or they were skeptical about its misapplication as a clinical decision tool [10, 11]. The Sepsis-3 Task Force itself has strongly encouraged independent validation in multiple health care settings to confirm its robustness and suggested that qSOFA should also be evaluated for outcomes other than mortality and ICU stay [5, 6].

Having the above considerations into mind, we endeavored to evaluate the discriminatory capacity of qSOFA versus SIRS criteria for predicting in-hospital mortality and ICU-free days in patients with suspected infection. In addition, we sought to assess the comparative accuracy of qSOFA and SIRS criteria for predicting other important clinical outcomes, such as ventilator-free days and organ dysfunction-free days.

## Methods

### Study setting and population

The Weill Cornell Medicine Registry and Biobank of Critically Ill Patients was initiated in October 2014 as an ongoing prospective cohort of critically ill adult (≥18 years old) patients admitted to the New York-Presbyterian Hospital-Weill Cornell Medical Center. All patients admitted to the medical ICU and willing to provide biological samples for research purposes are eligible for enrollment in the Registry and Biobank, unless they are cognitively impaired, unable to provide informed consent (or an appropriate legal representative cannot not be found to provide consent), admitted to the hospital purely to facilitate comfort care or unwilling to receive blood transfusion. Patients with a hemoglobin level of <7 g/dL upon admission are not eligible for the Registry and Biobank. In addition, for the current observational study, individuals who were transferred from an outside hospital and those admitted directly from the operating room were excluded because information on vital signs was lacking or might be affected by the surgery, respectively. Finally, only patients with suspicion of infection were considered for inclusion in the present study.

From patients included in the Weill Cornell Medicine Registry and Biobank, various biological samples (namely, plasma, blood cells, bronchoalveolar lavage fluid, urine, and cerebrospinal fluid) along with extensive clinical information (including vital signs before and during ICU hospitalization) are prospectively collected by physicians. Collected clinical data are stored using Research Electronic Data Capture (REDCap) electronic data capture tools [12] and are subsequently adjudicated by additional physicians.

### Assessment of qSOFA and SIRS

The qSOFA score included systolic blood pressure of ≤100 mmHg, respiratory rate of ≥22/minute, and altered mental status. The latter was not confined to a Glasgow Coma Scale score of <15, but it included any altered mentation, such as disorientation and somnolence [6]. One point was awarded for each of the above conditions and the score ranged from 0 to 3, as proposed by Seymour and colleagues [6].

SIRS score included temperature of >38 °C or <36 °C, heart rate of >90 beats/minute, respiratory rate of >20 breaths/minute, and white blood cell count of >12,000/mm^3^ or <4000/mm^3^ or >10% immature forms (bands). One point was awarded for each of the above conditions and the score ranged from 0 to 4, as proposed by Bone and colleagues [1].

Assessment of qSOFA and SIRS was done within 8 hours before ICU admission. The maximum score during that time window was recorded. Only acute changes from baseline were taken into account while calculating the scores. For example, a patient with known chronically altered mentation (e.g., due to an underlying neurological disease) was not given one point for “altered mental status” when his/her qSOFA score was calculated. This approach differs from that of Seymour and colleagues, who awarded a point to any patient with an abnormal mental status, not only those patients in whom abnormality reflected a change from baseline [6]. A sensitivity analysis was carried out by following the above approach of Seymour and colleagues [6].

Patients were categorized according to whether they had signs meeting two or more (qSOFA-positive) or less than two (qSOFA-negative) qSOFA criteria [6]. They were also categorized according to whether they met two or more (SIRS-positive) or less than two (SIRS-negative) SIRS criteria [3].

### Definition of suspicion of infection

Suspicion of infection was defined as clinical documentation to that effect (based on clinical presentation and radiological/laboratory findings) by the attending physician (in the emergency department, the hospital ward, or within the first day of ICU admission) and the subsequent administration of antimicrobials. This approach for defining suspicion of inspection, which requires documentation of the attending physician, differs from that of Seymour and colleagues, who relied solely upon the combination of antimicrobials and body fluid cultures [6]. A sensitivity analysis was carried out by following the definition of suspicion of infection used by Seymour and colleagues [6].

After the hospitalization of a patient was over, the medical charts were reviewed and the presence of infection was adjudicated on the basis of clinical context (such as response to antimicrobials), microbiological findings, and radiological studies. A subgroup analysis was carried out by including only patients with adjudicated infection.

### Study outcomes

All-cause in-hospital mortality was the primary outcome. ICU-free days from ICU admission to day 28, and ventilator-free days from initiation of invasive mechanical ventilation to day 28 served as secondary outcomes. Any organ dysfunction-free days and renal dysfunction-free days from ICU admission to day 14 were also considered secondary outcomes for this study.

ICU-free days were a composite outcome of mortality and length of ICU stay; patients who died in the ICU were considered to have zero ICU-free days. Similarly, ventilator-free days were defined as the number of days until day 28 without the need for invasive mechanical ventilation. Days after death were not considered as ventilator-free days.

With regard to organ dysfunction-free days, enrolled individuals were monitored for cardiovascular, respiratory, renal, neurological, coagulation, and hepatic dysfunction for 14 days after ICU admission. Cardiovascular dysfunction was defined as a systolic blood pressure of ≤90 mm Hg or need for vasopressors; respiratory dysfunction as a PaO_2_/FiO_2_ ratio of ≤300; coagulation dysfunction as a platelet count of ≤80,000/mm^3^; hepatic dysfunction as a serum bilirubin concentration of ≥2 mg/dL; neurological dysfunction as a Glasgow Coma Scale score of ≤12; and renal dysfunction as a serum creatinine concentration of ≥2 mg/dL [13]. The outcome of organ dysfunction-free days, by combining both the onset of organ dysfunction and its duration, provides more information than the onset of organ dysfunction alone. Also, this outcome takes mortality into account; days after death were not considered as organ dysfunction-free days [14].

### Data analysis and statistical methods

Categorical variables were presented as percentages and compared with the Fisher’s exact or chi-square test. Continuous variables were presented as median [interquartile range (IQR)] and compared with the nonparametric Mann-Whitney *U* test. Sensitivity, specificity, and the area under the receiver operating characteristic curve (AUC) with 95% confidence intervals (CI) were calculated for each score (namely, qSOFA and SIRS). For calculation of AUC, clinical outcomes other than in-hospital mortality (namely, ICU-free days, ventilator-free days, any organ dysfunction-free days, and renal dysfunction-free days) were considered as categorical variables with the median of the entire cohort serving as the threshold. For example, given that the median of ICU-free days of the entire cohort was 22 days, a threshold of 22 was used, and the discrimination of ICU-free days of >22 for each score was subsequently calculated. The Hanley and McNeil method was used for comparison of AUCs [15]. All analyses were carried out and relevant figures were made using GraphPad Prism 5.01 (GraphPad Software Inc., La Jolla, CA, USA) and MedCalc 16.8 (MedCalc Software bvba, Ostend, Belgium). A two-tailed *p* value of less than 0.05 denoted statistical significance.

## Results

One hundred fifty-two patients (82% of those enrolled in the Weill Cornell Medicine Registry and Biobank) had suspicion of infection and therefore were included in this study. Reasons of exclusion of the remaining subjects are detailed in Additional file 1.

Baseline characteristics and clinical outcomes of included patients are presented in Table 1. Forty-one per cent of individuals presented with pneumonia upon ICU admission and 45% had underlying malignancy. Infection was microbiologically confirmed in 67% and bacteremia was found in 37% of included subjects. The in-hospital mortality of the entire cohort was 19%.

**Table 1:** Baseline characteristics and clinical outcomes of included patients

Variable	All patients (*n* = 152)	Emergency department (*n* = 102)	Hospital wards (*n* = 50)	*p* value
Age	64 (51–75)	64 (48–76)	64 (53–71)	0.73
Female	69 (45)	51 (50)	18 (36)	0.12
Race				
White	98 (64)	64 (63)	34 (68)	0.59
Black	17 (11)	12 (12)	5 (10)	1.00
Hispanic	23 (15)	18 (18)	5 (10)	0.24
Other	14 (9)	8 (8)	6 (12)	0.56
Medical history				
Heart disease	40 (26)	31 (30)	9 (18)	0.11
Diabetes mellitus	26 (17)	19 (19)	7 (14)	0.64
COPD	10 (7)	9 (9)	1 (2)	0.17
CKD	29 (19)	19 (19)	10 (20)	0.83
Malignancy, any	69 (45)	36 (35)	33 (66)	0.0005
Malignancy, hematologic	43 (28)	18 (18)	25 (50)	<0.0001
Immunosuppression	66 (43)	36 (35)	30 (60)	0.005
Pneumonia	62 (41)	40 (39)	22 (44)	0.60
Acute kidney injury^a^	86 (57)	61 (60)	25 (50)	0.29
APACHE II score	25 (18–31)	25 (17–30)	26 (20–33)	0.08
Positive cultures	102 (67)	64 (63)	38 (76)	0.14
Confirmed bacteremia	38 (25)	26 (25)	12 (24)	1.00
Adjudicated infection^b^	133 (88)	89 (87)	44 (88)	1.00
Vasopressors in patients with adjudicated infection	68 (45)	45 (44)	23 (46)	0.86
ARDS^c^	16 (11)	9 (9)	7 (14)	0.4
In-hospital mortality	29 (19)	12 (12)	17 (34)	0.002
ICU-free days^d^	22 (14–25)	23 (19–25)	20 (0–24)	0.03
Ventilator-free days^e^	28 (20–28)	28 (23–28)	23 (18–28)	0.002
Any organ dysfunction-free days^f^	4 (0–11)	9 (0–11)	0 (0–5)	0.001
Renal dysfunction-free days^f^	13 (7–14)	14 (9–14)	12 (2–14)	0.15

Data are presented as median (interquartile range) or number (%) and compared with the Mann Whitney *U* test or the Fisher’s exact test, respectively

*Abbreviations: COPD* chronic obstructive pulmonary disease, *CKD* chronic kidney disease, *APACHE II* Acute Physiology and Chronic Health Evaluation II, *ARDS* acute respiratory distress syndrome, *ICU* intensive care unit

^a^Defined as an increase in serum creatinine of 0.3 mg/dL or >50% from baseline

^b^On the basis of clinical context, microbiological findings, and radiological studies

^c^Defined according to the Berlin definition

^d^From ICU admission to day 28

^e^From initiation of invasive mechanical ventilation to day 28

^f^From ICU admission to day 14

One hundred and two (67%) included patients were admitted to the medical ICU within less than 24 hours after their presentation in the emergency department, while the remaining 23% were hospitalized in the wards for 24 hours or more before their transfer to the ICU (Additional file 1). Compared to patients from the emergency department, patients from the hospital wards were more likely to have underlying malignancy (*p* < 0.001) and be immunosuppressed (*p* < 0.01) (Table 1). Patients from hospital wards had higher in-hospital mortality (*p* < 0.01), fewer ICU-free days (*p* = 0.03), fewer ventilator-free days (*p* < 0.01), and fewer any organ dysfunction-free days (*p* < 0.01) than those coming from the emergency department (Table 1).

Of the included patients, 36% were qSOFA-negative. Distribution of signs meeting qSOFA criteria in included patients is summarized in Additional file 2.

### In-hospital mortality

**Fig. 1:**
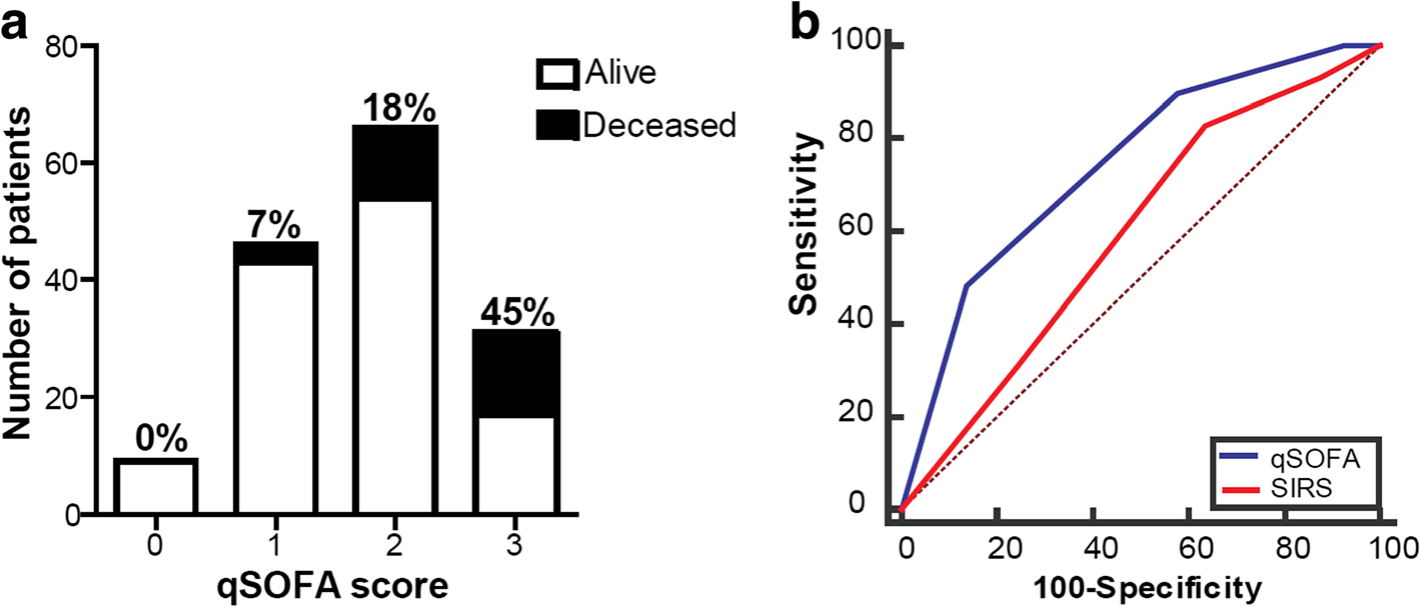
Association between in-hospital mortality and qSOFA calculated within 8 hours before ICU admission in patients with suspected infection. **a** Distribution of included patients according to number of qSOFA criteria met and corresponding mortality rates (*p* < 0.001 using chi-square test). **b** Comparison of the area under the receiver operating characteristic curves of qSOFA and SIRS criteria for in-hospital mortality (*p* = 0.03 using the Hanley and McNeil method). *Abbreviations*: *qSOFA* quick Sequential (Sepsis-related) Organ Failure Assessment, *SIRS* Systemic Inflammatory Response Syndrome, *ICU* intensive care unit

In-hospital mortality of qSOFA-positive patients was higher than that of qSOFA-negative patients (27% vs 6%; *p* < 0.01). In-hospital mortality of patients with zero, one, two, or three qSOFA criteria was 0%, 7%, 18%, and 45%, respectively (*p* < 0.001) (Fig. 1a). The discrimination of in-hospital mortality using qSOFA (AUC, 0.74; 95% CI, 0.66–0.81) was significantly greater compared with SIRS criteria (AUC, 0.59; 95% CI, 0.51–0.67; *p* = 0.03) (Fig. 1b).

Sensitivity and specificity at different thresholds for qSOFA and SIRS are summarized in Table 2. A qSOFA score greater than or equal to two had a 90% sensitivity and 42% specificity for in-hospital mortality compared to 93% sensitivity and 12% specificity for SIRS greater than or equal to two (Table 2).

**Table 2:** Sensitivity and specificity at different thresholds for qSOFA and SIRS for various clinical outcomes of included patients

Variable	qSOFA			SIRS			
	≥1	≥2	≥3	≥1	≥2	≥3	≥4
In-hospital mortality							
Sensitivity, % (95% CI)^a^	100 (88–100)	90 (73–98)	48 (29–68)	100 (88–100)	93 (77–99)	83 (64–92)	31 (15–51)
Specificity, % (95% CI)^a^	7 (3–13)	42 (33–52)	86 (79–92)	0 (0–3)	12 (7–19)	37 (28–46)	76 (67–83)
ICU-free days^b^							
Sensitivity, % (95% CI)	99 (92–100)	76 (65–86)	28 (18–40)	100 (95–100)	92 (83–97)	71 (59–81)	26 (17–38)
Specificity, % (95% CI)	10 (4–18)	48 (36–59)	86 (77–93)	0 (0–5)	14 (7–23)	36 (26–48)	75 (64–84)
Ventilator-free days^b^							
Sensitivity, % (95% CI)	99 (93–100)	74 (62–83)	29 (19–41)	100 (95–100)	93 (85–98)	72 (60–82)	29 (19–41)
Specificity, % (95% CI)	10 (4–19)	45 (34–57)	88 (78–94)	0 (0–5)	15 (8–25)	38 (27–49)	78 (67–86)
Any organ dysfunction-free days^b^							
Sensitivity, % (95% CI)	96 (89–99)	68 (57–79)	28 (18–39)	100 (95–100)	89 (80–95)	68 (57–79)	25 (16–36)
Specificity, % (95% CI)	8 (3–16)	41 (30–53)	87 (77–94)	0 (0–5)	12 (6–21)	34 (24–46)	74 (62–83)
Renal dysfunction-free days^b^							
Sensitivity, % (95% CI)	95 (87–99)	70 (58–80)	26 (17–38)	100 (95–100)	88 (79–94)	68 (57–79)	26 (17–38)
Specificity, % (95% CI)	7 (2–15)	42 (31–54)	86 (76–93)	0 (0–5)	11 (5–20)	34 (24–46)	75 (64–84)

*Abbreviations: qSOFA* quick Sequential (Sepsis-related) Organ Failure Assessment, *SIRS* Systemic Inflammatory Response Syndrome, *ICU* intensive care unit, *CI* confidence intervals

^a^Sensitivity was calculated on the basis of the number of participants who experienced the clinical outcome. Specificity was calculated on the basis of the number of participants who did not experience the clinical outcome

^b^Clinical outcomes other than in-hospital mortality (namely, ICU-free days, ventilator-free days, any organ dysfunction-free days, and renal dysfunction-free days) were considered as categorical variables with the median of the entire cohort serving as the threshold. The median of the entire cohort for ICU-free days, ventilator-free days, any organ dysfunction-free days, and renal dysfunction-free days was 22, 28, 5, and 14 days, respectively. Thus, the sensitivity and specificity for ICU-free days <22, ventilator-free days <28, any organ dysfunction-free days <5, and renal dysfunction-free days <14 were calculated

#### Subgroup analyses

In the subgroup of patients with adjudicated infection, the discrimination of in-hospital mortality using qSOFA (AUC, 0.73; 95% CI, 0.65–0.81) was significantly greater compared with SIRS criteria (AUC, 0.57; 95% CI, 0.48–0.66; *p* = 0.03).

In the subgroup of patients with adjudicated infection who required vasopressors, the discrimination of in-hospital mortality using qSOFA (AUC, 0.69; 95% CI, 0.57–0.80) was also greater, albeit statistically nonsignificant, compared with SIRS criteria (AUC, 0.52; 95% CI, 0.40–0.65; *p* = 0.07).

#### Sensitivity analyses

The superior discriminatory capacity of qSOFA over SIRS criteria was maintained even when the approach of Seymour and colleagues was followed for defining altered mental status and suspicion of infection [6]. In detail, the discrimination of in-hospital mortality using qSOFA (measured in accordance with Seymour and colleagues for altered mentation) (AUC, 0.73; 95% CI, 0.65–0.80) was greater compared with SIRS criteria (AUC, 0.59; 95% CI, 0.51–0.67; *p* = 0.046) [6]. Similarly, the discrimination of in-hospital mortality using qSOFA (AUC, 0.75; 95% CI, 0.67–0.82) was greater compared with SIRS criteria (AUC, 0.58; 95% CI, 0.49–0.66; *p* = 0.02) even when suspicion of infection was defined according to the original qSOFA publication [6].

Finally, the performance of qSOFA to predict mortality was compared with the previous definition of severe sepsis, namely a SIRS score ≥ 2 plus evidence of organ dysfunction or blood lactate level > 2 mmoL/L [16]. The discrimination of in-hospital mortality using qSOFA (AUC, 0.74; 95% CI, 0.66–0.81) was greater compared with the previous definition of severe sepsis (AUC, 0.57; 95% CI, 0.49–0.65; *p* = 0.01).

### ICU-free days and ventilator-free days

**Fig. 2:**
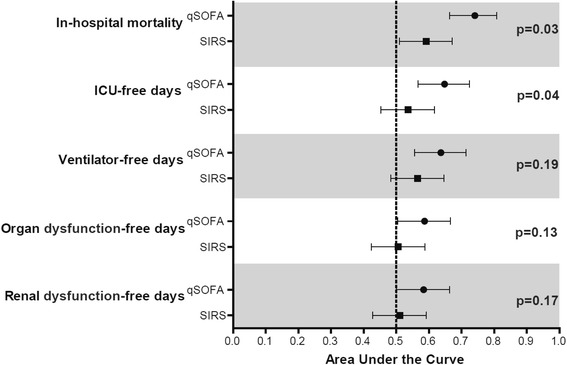
Comparison of the area under the receiver operating characteristic curves of qSOFA and SIRS for important clinical outcomes of patients with suspected infection outside the ICU and corresponding *p* values using the Hanley and McNeil method. Clinical outcomes other than in-hospital mortality (namely, ICU-free days, ventilator-free days, any organ dysfunction-free days, and renal dysfunction-free days) were considered as categorical variables with the median of the entire cohort serving as the threshold. The median of the entire cohort for ICU-free days, ventilator-free days, any organ dysfunction-free days, and renal dysfunction-free days was 22, 28, 5, and 14 days, respectively. Thus, the area under the receiver operating characteristic curve and 95% confidence intervals for ICU-free days <22, ventilator-free days <28, any organ dysfunction-free days <5, and renal dysfunction-free days <14 were calculated and displayed in this figure. *Abbreviations: qSOFA* quick Sequential (Sepsis-related) Organ Failure Assessment, *SIRS* Systemic Inflammatory Response Syndrome, *ICU* intensive care unit

ICU-free days of qSOFA-positive patients were fewer than qSOFA-negative patients [median, 20 days (IQR, 6–24) vs 24 days (IQR, 21–25); *p* < 0.001]. The discrimination of ICU-free days <22 (i.e., <median of the entire cohort) using qSOFA (AUC, 0.65; 95% CI, 0.57–0.72) was significantly greater compared with SIRS criteria (AUC, 0.54; 95% CI, 0.45–0.62; *p* = 0.04) (Fig. 2).

Ventilator-free days of qSOFA-positive patients were fewer than qSOFA-negative patients [median, 26 days (IQR, 13–28) vs 28 days (IQR, 24–28); *p* < 0.01]. The discrimination of ventilator-free days <28 using qSOFA (AUC, 0.64; 95% CI, 0.56–0.71) was not different from that using SIRS criteria (AUC, 0.57; 95% CI, 0.48–0.65; *p* = 0.19) (Fig. 2).

### Any organ dysfunction-free days and renal dysfunction-free days

There was no difference between qSOFA-positive and qSOFA-negative patients in terms of organ dysfunction-free days; both any organ dysfunction [median, 3 days (IQR, 0–11) vs 9 days (IQR, 0–12); *p* = 0.12] and specifically renal dysfunction [median, 12 days (IQR, 4–14) vs 14 days (IQR, 10–14); *p* = 0.07]. Consistently, there was no difference between qSOFA and SIRS criteria in predicting any organ dysfunction-free days <5 (AUC, 0.59; 95% CI, 0.51–0.67 vs AUC, 0.51; 95% CI, 0.42–0.59; *p* = 0.13) and renal dysfunction-free days <14 (AUC, 0.58; 95% CI, 0.50–0.66 vs AUC, 0.51; 95% CI, 0.43–0.59; *p* = 0.17) (Fig. 2).

For all secondary outcomes, sensitivity and specificity at different thresholds for qSOFA and SIRS criteria are summarized in Table 2.

## Discussion

The results of the present study suggest that qSOFA is more accurate than SIRS for predicting in-hospital mortality and ICU-free days, but not ventilator-free days, any organ dysfunction-free days or renal dysfunction-free days.

The finding from our well-phenotyped cohort of critically ill patients, that qSOFA predicts mortality better than SIRS, corroborates the publication by Seymour and colleagues [6]. This finding is also in line with another recently published large retrospective study [17]. By analyzing data from electronic health records, Churpek and colleagues showed that qSOFA performed better than SIRS for predicting in-hospital mortality [17]. However, accuracy of qSOFA was worse than that of general early warning scores, such as the Modified Early Warning Score and the National Warning Score [17]. Discrimination of mortality using qSOFA was also lower than the Mortality in Emergency Department Sepsis (MEDS) score according to another retrospective study [18]. The latter study by Wang and colleagues did not compare qSOFA with SIRS score [18]. Thus, our report may contribute to the accumulating evidence on the potential clinical usefulness of qSOFA.

Our main finding, that qSOFA predicts mortality more accurately than SIRS criteria, was maintained in the subgroup and sensitivity analyses that we carried out. Interestingly, discrimination of in-hospital mortality using qSOFA was greater than the previous definition of severe sepsis, namely a SIRS score ≥ 2 plus evidence of organ dysfunction or blood lactate level > 2 mmoL/L [16]. The latter finding is in line with the recently published international prospective cohort study by Freund and colleagues, who reported that qSOFA performed better than severe sepsis for predicting mortality [19]. Freund and colleagues defined severe sepsis as the sole combination of SIRS ≥ 2 plus hyperlactatemia, without taking into account other evidence of organ dysfunction [19]. One could support that the comparison between qSOFA and the previous definition of severe sepsis (which presumably had high specificity for mortality) is more proper than the comparison between qSOFA and SIRS score (which was intended to be a sensitive but not specific sign for predicting mortality) [1, 16].

Our main finding, that qSOFA predicts mortality better than SIRS criteria, was derived from comparison of AUCs. It should be emphasized that although AUCs are good to show that a test has overall better discriminatory capacity than another test, the real features of interest are the sensitivity and specificity of a given cutoff point, which is proposed for clinical use (e.g., for SIRS this point was two). In Table 2, it is showed that a SIRS score ≥ 3 would be a better discriminator than SIRS ≥ 2 for in-hospital mortality. In Table 2, we provided the sensitivity and specificity at different cutoff points of qSOFA and SIRS criteria for all outcomes.

To the best of our knowledge, our study is the first to compare the predictive accuracy of qSOFA versus SIRS criteria for outcomes other than mortality and ICU stay. The authors of the original qSOFA publication acknowledged it as a limitation that they focused to only two outcomes (namely, mortality and ICU stay) and they advocated for research on other outcomes [6]. We found that qSOFA was not better than SIRS criteria for predicting adverse events other than mortality and ICU-free days, namely ventilator-free days, any organ dysfunction free-days, and renal dysfunction-free days. Although it could not be precluded that our study was not big enough to reveal a difference in such outcomes, another plausible explanation may be that qSOFA does not take into account signs of such organ failures as renal failure, hepatic failure, hypoxemia, or coagulopathy [20]. Even proponents of qSOFA hinted at its potential weakness to capture forms of organ failure different than those assessed using qSOFA [7]. Future research is needed to confirm or refute this interesting finding.

Our results seem to justify the concern that qSOFA may be less sensitive (albeit more specific) than SIRS for predicting clinical deterioration in patients at risk of sepsis [9, 17]. Indeed, we found that qSOFA ≥2 had a 76% sensitivity for ICU-free days compared to 92% for SIRS ≥2. The same applied for outcomes, such as ventilator-free days (74% versus 93%), any organ dysfunction-free days (68% versus 89%), and renal dysfunction-free days (70% versus 88%) (Table 2). It has been supported that the high sensitivity of SIRS may make its usage for screening of sepsis impractical, because it identifies many patients who are likely to have normal regulated responses as opposed to the dysregulated response that defines sepsis [4]. On the other hand, 6% of qSOFA-negative patients in our cohort died in the hospital. All those qSOFA-negative patients had one point suggesting that a negative qSOFA score, especially if borderline (i.e., qSOFA score of one) and combined with a positive SIRS score, might not be reassuring. A similar conclusion was reached by Churpek and colleagues, who found that half of their study patients did not meet ≥2 qSOFA criteria at the time of their death or ICU transfer [17]. In contrast, Freund and colleagues showed a very low mortality rate of qSOFA-negative patients and they therefore inferred that qSOFA could replace SIRS without the risk of missing critically ill patients [19].

A comparative strength of our study is the collection of extensive clinical information, which allows us to assess whether the observed altered sign may have an explanation other than infection, and to distinguish between acute and chronic conditions. Previous relevant studies were limited by the fact that “no organ dysfunction measurements evaluated [by them] distinguish between chronic and acute organ dysfunction”, as acknowledged by their authors [6]. Thus, our findings complement those derived from previous relevant studies, which were based on large electronic health record databases [6, 17].

Our study has certain limitations. First, in accordance with the original qSOFA publication [6], we chose all-cause mortality (instead of sepsis-related mortality) and patients with suspicion of sepsis (instead of all critically ill patients) as our study outcome and population, respectively. Second, due to the design of our Registry and Biobank, we could not measure qSOFA and SIRS scores earlier than 8 hours before ICU admission and we could not specify the exact timing that the above scores became positive. Thus, we were not able to evaluate the interesting finding by Churpek and colleagues, who reported that most patients met ≥2 SIRS criteria 17 hours prior to the adverse event of ICU transfer or death compared to 5 hours for ≥2 qSOFA criteria [17]. Third, although we and others [6, 17, 19, 21] compared qSOFA with SIRS score, these scores are not mutually exclusive; indeed, 93 (61%) patients in our cohort met concurrently ≥2 qSOFA and ≥2 SIRS criteria. Fourth, one could wonder whether our database did not include the most severe patients (i.e., those who were more likely to have higher qSOFA score), given that we inevitably excluded patients from whom informed consent could not be obtained. However, the in-hospital mortality of our cohort (19%) was identical to that (19%) of Raith and colleagues, which also involved subjects with suspected infection requiring admission to the ICU [21]; a fact which may indicate that our cohort is representative of such a patient population.

Finally, all included patients were eventually admitted to the ICU and therefore our study differs from the original qSOFA publication in that ICU transfer could not serve as its outcome [6]. The decision for ICU transfer depends on the availability of ICU beds and varies across countries [22]. Also, the nature of our dataset precluded determining if either score identified patients who, despite suspected infection, were not admitted to the ICU; indeed, patients with low qSOFA and/or low SIRS score might not be admitted to the ICU and subsequently they would not be included in our analysis. Taken together, our study population was more selected (i.e., more likely to have poor prognosis) compared to those of previous relevant studies [6, 17, 19]. However, we calculated qSOFA and SIRS criteria while the patients were still outside the ICU and before the initiation of interventions (such as sedation, mechanical ventilation, and vasopressors) which affect the scores.

## Conclusions

In conclusion, our findings suggest that the newly introduced qSOFA provides better discrimination than SIRS for predicting mortality and ICU-free days. However, it may be less clear whether qSOFA is also better than SIRS criteria for predicting ventilator-free days and organ dysfunction-free days. These findings may help clinicians gain further insight into the usefulness of qSOFA.

## Key messages

- In patients with suspected infection who eventually required admission to the ICU, qSOFA calculated before their ICU admission provided better discrimination than SIRS criteria for predicting mortality and ICU-free days.
- It may be less clear whether qSOFA is better than SIRS criteria for predicting ventilator-free days and organ dysfunction-free days.
- These findings may help clinicians gain further insight into the usefulness of qSOFA.

## Additional files

Additional file 1: Flow diagram of patients included in the study. (DOCX 26 kb)

Additional file 2: Distribution of components of qSOFA in patients included in the study. (DOCX 14 kb)

## Abbreviations

APACHE II: Acute Physiology and Chronic Health Evaluation II
ARDS: Acute respiratory distress syndrome
AUC: Area under the receiver operating characteristic curve
CI: Confidence interval
CKD: Chronic kidney disease
COPD: Chronic obstructive pulmonary disease
ICU: Intensive care unit
IQR: Interquartile range
qSOFA: quick Sequential (Sepsis-related) Organ Failure Assessment
REDCap: Research Electronic Data Capture
SIRS: Systemic Inflammatory Response Syndrome
SOFA: Sequential (Sepsis-related) Organ Failure Assessment
