# Supplementary material for: Comparison of qSOFA and SIRS for predicting adverse outcomes of patients with suspicion of sepsis outside the intensive care unit
Source: Crit Care. 2017 Mar 26;21:73. doi: 10.1186/s13054-017-1658-5 (PMC5366240; doi:10.1186/s13054-017-1658-5)
Supplement: Supplementary file 1 — Flow diagram of patients included in the study. (DOCX 26 kb) [file 13054_2017_1658_MOESM1_ESM.docx]

**ADDITIONAL FILE 1**

**Supplemental Figure. Flow diagram of patients included in the study.**

^1^ Suspicion of infection was defined as clinical documentation to that effect by the attending physician and the subsequent administration of antimicrobials.

^2^ One hundred and two included patients were admitted to the medical intensive care unit within less than 24 hours after their presentation in the emergency department, while the remaining 50 patients were hospitalized in the hospital wards for 24 hours or more before their transfer to the medical intensive care unit.
