# Supplementary material for: Comparison of qSOFA and SIRS for predicting adverse outcomes of patients with suspicion of sepsis outside the intensive care unit
Source: Crit Care. 2017 Mar 26;21:73. doi: 10.1186/s13054-017-1658-5 (PMC5366240; doi:10.1186/s13054-017-1658-5)
Supplement: Supplementary file 2 — Distribution of components of qSOFA in patients included in the study. (DOCX 14 kb) [file 13054_2017_1658_MOESM2_ESM.docx]

**ADDITIONAL FILE 2**

| **Supplemental Table. Distribution of components of qSOFA in patients included in the study.** | | | |
| --- | --- | --- | --- |
| **Variable** | **All patients (n=152)** | **Patients with positive qSOFA score**  **(n=97)** | **Patients with negative qSOFA score**  **(n=55)** |
| **qSOFA criterion met,  n (%)^1^** |  |  |  |
| Systolic blood pressure  ≤100 mmHg | 98 (64) | 86 (89) | 12 (22) |
| Respiratory rate  ≥22/min | 119 (78) | 89 (92) | 30 (55) |
| Altered mental status | 54 (36) | 49 (56) | 5 (9) |
| **Number of criteria met^2^** |  |  |  |
| Median | 2 | 2 | 1 |
| Interquartile range | 1-2 | 2-3 | 1-1 |
| Distribution |  |  |  |
| 0 | 9 (6) | 0 | 9 (16) |
| 1 | 46 (30) | 0 | 46 (84) |
| 2 | 66 (43) | 66 (68) | 0 |
| 3 | 31 (20) | 31 (32) | 0 |

*Abbreviation:* qSOFA: quick Sequential (Sepsis-related) Organ Failure Assessment.

^1^ Patients may have more than one criterion.

^2^ Summary of percentages may not equal 100% due to rounding.
